# NMR-TS: de novo molecule identification from NMR spectra

**DOI:** 10.1080/14686996.2020.1793382

**Published:** 2020-07-30

**Authors:** Jinzhe Zhang, Kei Terayama, Masato Sumita, Kazuki Yoshizoe, Kengo Ito, Jun Kikuchi, Koji Tsuda

**Affiliations:** a Department of Computational Biology and Medical Sciences, Graduate School of Frontier Sciences, The University of Tokyo, Kashiwa, Japan; b RIKEN Center for Advanced Intelligence Project, Tokyo, Japan; c Graduate School of Medicine, Kyoto University, Kyoto, Japan; d RIKEN Medical Sciences Innovation Hub Program (MIH), Yokohama, Japan; e Graduate School of Medical Life Science, Yokohama City University, Yokohama, Japan; f International Center for Materials Nanoarchitectonics (WPI-MANA), National Institute for Materials Science, Tsukuba, Japan; g RIKEN Center for Sustainable Resource Science, Yokohama, Japan; h Graduate School of Bioagricultural Sciences, Nagoya University, Nagoya, Japan; i Research and Services Division of Materials Data and Integrated System, National Institute for Materials Science, Tsukuba, Japan

**Keywords:** NMR, deep learning, molecule generation, density functional theory, 404 Materials informatics / Genomics

## Abstract

Nuclear magnetic resonance (NMR) spectroscopy is an effective tool for identifying molecules in a sample. Although many previously observed NMR spectra are accumulated in public databases, they cover only a tiny fraction of the chemical space, and molecule identification is typically accomplished manually based on expert knowledge. Herein, we propose NMR-TS, a machine-learning-based python library, to automatically identify a molecule from its NMR spectrum. NMR-TS discovers candidate molecules whose NMR spectra match the target spectrum by using deep learning and density functional theory (DFT)-computed spectra. As a proof-of-concept, we identify prototypical metabolites from their computed spectra. After an average 5451 DFT runs for each spectrum, six of the nine molecules are identified correctly, and proximal molecules are obtained in the other cases. This encouraging result implies that de novo molecule generation can contribute to the fully automated identification of chemical structures. NMR-TS is available at https://github.com/tsudalab/NMR-TS.

## Graphical abstract

**Figure:**
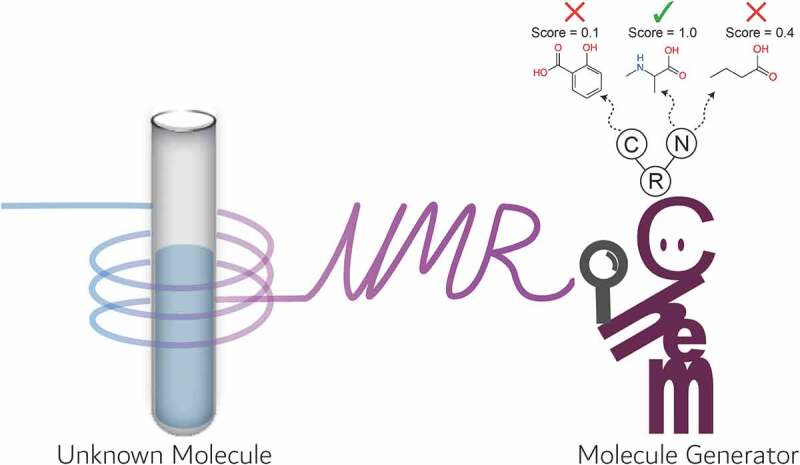

## 1. Introduction

Nuclear magnetic resonance (NMR) spectroscopy is indispensable for chemists for various chemical structure identification tasks, such as confirming the synthesis of a molecule [1] and revealing the existence of impurities [2–4]. An NMR spectrum consists of peaks that correspond to molecular fragments, and the peak positions (chemical shifts) depend on the environment in the molecule. One important task for chemists is peak assignment, in which they use their knowledge to map peaks to functional groups depending on their chemical shifts. The approximate chemical structure of the target molecule can then be confirmed from the NMR spectrum.

Recently, automated robotic laboratory systems have received considerable attention for high-throughput material design. Automated robotic laboratories perform chemical reactivity tests under different reaction conditions guided by machine learning algorithms [5–7]. A key step in this automated experiment is the qualification and quantification of the reaction products from each cycle to evaluate the reactivity. NMR spectroscopy can be used as a method for either qualitative or quantitative measurements. In automated robotic laboratory system studies, NMR spectroscopy has already been applied for quantitative measurements [7]. Automating the identification of any molecule would pave the way for using NMR spectroscopy for the qualitative measurement of reaction products in robotic laboratory systems.

To date, predicting the molecules in a sample from its NMR spectrum has mainly been performed based on databases [8–10]. For example, to identify the metabolites in a sample, methods for accurately calculating chemical shifts [11,12] and for predicting molecules using specific databases [13–16] have been proposed. However, these methods are not effective for unknown molecules whose spectra are not registered in the database. In addition, even for prediction methods based on computational chemistry [17] and machine learning [18–20], NMR spectra cannot be calculated without the structures of the compounds; thus, these methods are also not useful for unknown molecules [21,22]. In this work, we try to identify unknown molecules from its NMR spectrum with a de novo molecule generator.

Recent progress in machine learning has enabled the development of de novo molecule generators [23–27, 28–30], which are expected to design molecules with desired properties [24]. For instance, we developed a molecule generator, ChemTS [27], which combines Monte Carlo tree search (MCTS) with a recurrent neural network (RNN), and successfully showed that ChemTS coupled with quantum chemical calculations can produce realistic molecules that have desired properties [31]. So far, most de novo molecule generators have only been tested or applied on quantifiable chemical properties such as gaps between the highest occupied molecular orbital (HOMO) and the lowest unoccupied molecular orbital (LUMO). As ^1^ H NMR spectra are highly characteristic of individual compounds, we consider ^1^ H NMR spectra as one of its molecular properties.

We developed a python library named NMR-TS to identify an unknown molecule from its spectrum by designing molecules that have ^1^ H NMR spectra that are as similar as possible to the target spectrum. In this work, as a proof-of-concept, we evaluated the ^1^ H NMR spectra of nine known molecules that were not included in the NMR-TS training set. NMR-TS succeeded in correctly identifying six of the nine molecules from their ^1^ H NMR spectra, whereas proximal molecules were obtained in the other three cases.

## 2. Methods

**Figure 1.**
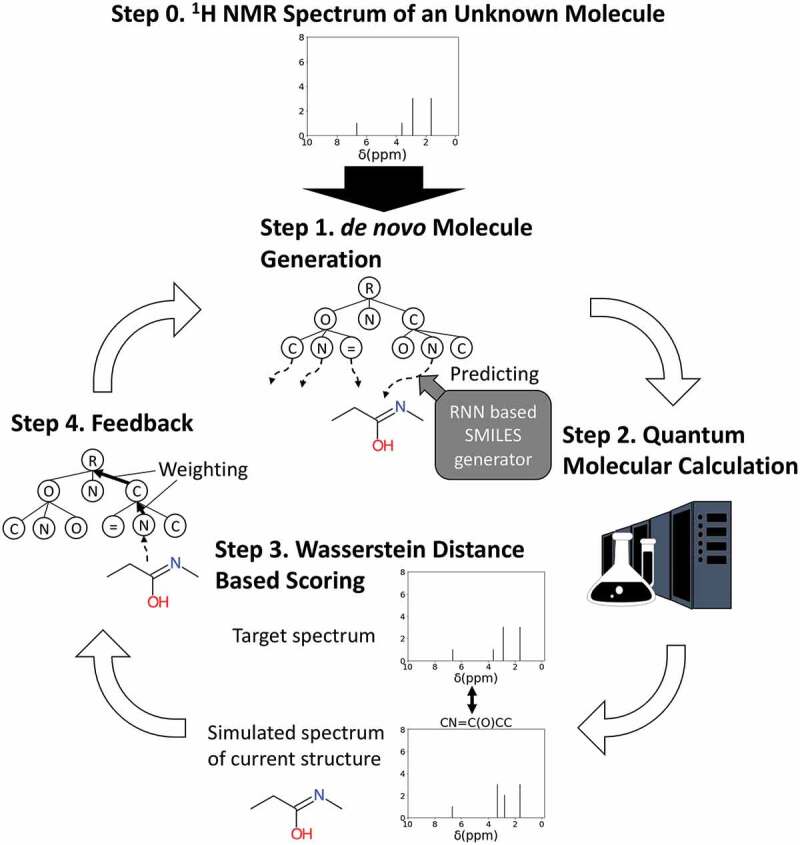
Concept of this study and molecular generator scheme. NMR-TS tries to identify an unknown molecule from its NMR spectrum (target NMR spectrum) by designing molecules with NMR spectra as similar as possible to the target NMR spectrum. The NMR spectrum of a generated molecule is simulated by quantum chemical calculation. The Wasserstein distance is used to quantify the proximity between the NMR spectra of the target and generated molecules.

NMR-TS is a tool that automatically identifies the molecular structure from a given NMR spectrum based on ChemTS. The NMR-TS method is schematized in Figure 1. NMR-TS requires (1) a target ^1^ H NMR spectrum, (2) the numbers of hydrogen and carbon atoms, which indicate the size of the target molecule, and (3) a training data set (a database of SMILES30 strings) as input. NMR-TS outputs a list of candidate molecular structures that fit the input spectrum.

### 2.1. ChemTS

Before describing NMR-TS, we introduce ChemTS, which is the base algorithm of NMR-TS. The input for ChemTS is a database of SMILES [32] strings and an evaluation function, which quantifies the goodness of a generated molecule. Starting from a root node that represents the beginning of a SMILES string, the ChemTS algorithm builds a search tree, in which each node corresponds to one SMILES symbol. The ChemTS search process consists of four procedures: selection, expansion, simulation, and backpropagation, the details of which are given in the original paper [27]. In the selection step, the tree is traversed from the root to a leaf by recursively choosing the child node that has the maximum upper confidence bound (UCB) based score at each branch. This score is described in detail in the next paragraph. A path from the root to the leaf node becomes a SMILES prefix. In the expansion step, several child nodes are added to the leaf node. Upon tree expansion, a selected prefix serves as the input for the RNN pretrained on the database. With the SMILES prefix as an input, the RNN can predict the next symbol after the prefix and elongate the length of the prefix by one. By repeating this elongation step until a terminal symbol appears, a complete SMILES string is generated [33]. The generated molecule is evaluated using the evaluation function and then the tree is updated accordingly during the backpropagation procedure. The input database for pretraining the RNN can be either a general database with no specific molecular characteristics or a specific database containing field-specific SMILES strings.

In this study, to perform the massive DFT computations, we parallelized the tree search part of ChemTS using Open MPI based on the virtual loss approach [34]. We used the following scoring in the selection step to avoid concentrating the DFT computations on one node.

(1) $$ucb_{i}=\frac{S_{i}}{v_{i}+w_{i}}+CP_{i}\frac{\sqrt{v_{p}+w_{p}}}{1+v_{i\;}+w_{i}}$$

Here, $s_{i}$ is the total score obtained by node i, $v_{i}$ is the total visit number of i, $w_{i}$ is the total virtual visit number of i (virtual loss), $v_{p}$ is the total visit number of parent node p of i, $w_{p}$ is the total virtual visit number of p, $p_{i}$ is the probability of i among the children of p, and C is a constant that controls the exploration–exploitation trade-off.

### 2.2. NMR-TS

Similar to ChemTS, NMR-TS pretrains an RNN model using the input SMILES database to obtain an RNN model that can generate various valid SMILES strings depending on the input prefixes. NMR-TS takes a target NMR spectrum as input (Step 0 in Figure 1). In the generation step of NMR-TS (Step 1 in Figure 1), a SMILES prefix determined by MCTS is given to the RNN model to obtain a complete SMILES string. Then, the simulated NMR spectrum of the SMILES string is computed using a quantum-molecular-calculation-based method (Step 2 in Figure 1), as described in the following section. Once the simulated NMR spectrum of the generated molecule is obtained, its similarity with the target NMR spectrum is evaluated using the Wasserstein distance (Step 3 in Figure 1). In addition, the numbers of hydrogen and carbon atoms in the target molecules are used to constrain the sizes of the molecules generated by NMR-TS. If the numbers of hydrogen or carbon atoms differ between the target and generated molecules, a penalty is added according to the difference. A score is calculated by integrating both the Wasserstein distance and the atom number penalty. The score of each prefix branch is updated using the calculated score to progress the MCTS (Step 4 in Figure 1). Once the search tree is updated, a new SMILES prefix is selected, and the above steps are repeated. By repeating these steps, we expect that the tree will eventually explore the chemical space and provide molecules that fit the target spectrum.

### 2.3. NMR spectrum prediction

To compute the ^1^ H NMR spectra, we started by converting the input SMILES string into the canonical SMILES format, which was converted into a 3D molecular structure [35] through the function implemented in the RDkit library [36] with the random seed fixed to 1. Canonizing the SMILES string and fixing the random seed ensured that identical chemical structures produce the same ^1^ H NMR spectrum in the prediction step. Once RDkit produced a molecular structure, with the atom positions described by Cartesian coordinates, the ^1^ H NMR spectrum was computed using density functional theory (DFT) [37] at the B3LYP/3-21 G* level on the optimized structure at the universal force field (UFF) level. Magnetic shielding tensors at the proton positions were calculated using the gauge-invariant atomic orbital (GIAO) method. The isotropic chemical shift in the ^1^ H NMR spectrum was estimated by subtracting that of tetramethylsilane (TMS) calculated at the same level. For temporal convenience, we ignored the degeneracy between protons in this work. Hence, the ^1^ H NMR spectrum of a molecule was computed as a line spectrum of all the protons in the molecule. All DFT calculations were performed with the Gaussian 16 package [38].

### 2.4. Wasserstein distance and evaluation function

**Figure 2.**
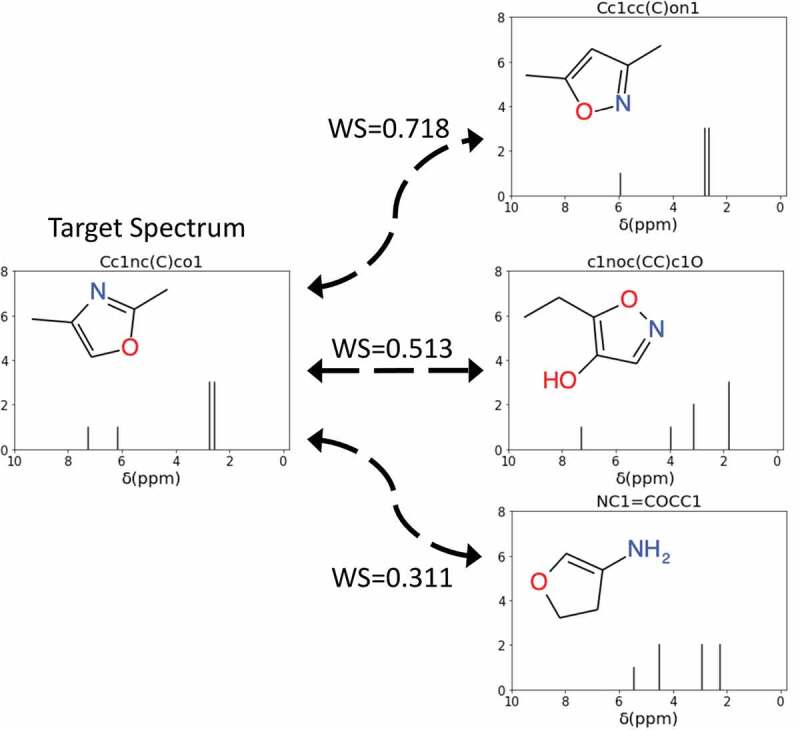
Examples of using the Wasserstein score (WS) to quantify the difference between the target NMR spectrum and the NMR spectra of SMILES generated molecules. A WS closer to 1 indicates high similarity between the spectra. In this example, the spectrum of Cc1cc(C)on1 is most similar to the target spectrum.

The Wasserstein distance [39], also known as the Kantorovich–Rubinstein metric or the earth mover’s distance [40], is a function that describes the distance between two distributions. If we consider two distributions as two piles of dirt, the Wasserstein distance is the minimum amount of work needed to reshape one into the other. Some typical examples of the distances between the calculated NMR spectra of molecules are shown in Figure 2. In this study, the Wasserstein distance was used to evaluate the difference between the NMR spectra of a newly generated SMILES string and the target NMR spectra. We also used information about the numbers of hydrogen and carbon atoms in our evaluation function to guide the MCTS. We defined the evaluation function $Score\left(M_{g},M_{t}\right)$ between a generated molecule $M_{g}$ and a target (unknown) molecule $M_{t}$ as follows:

(2) $$\begin{array}{rl} & Score\left(M_{g},M_{t}\right)=1-tanh\left(WD\left(M_{g},M_{t}\right)\right.\\ & \;\;\;\;\;\;\;\;\;\;\;\;\;\;\;\;\;\;\;\;\;\;\;\;\;\;\;\;\;\;\;\;\;\;\;\;\left.+\alpha Penalty\left(M_{g},M_{t}\right)\right) \end{array}$$

(3) $$Penalty\left(M_{g},M_{t}\right)=\left|C\left(M_{g}\right)-C\left(M_{t}\right)\right|+\left|H\left(M_{g}\right)-H\left(M_{t}\right)\right|$$

where $WD\left(M_{g},M_{t}\right)$ is the Wasserstein distance between the calculated ^1^ H NMR spectra of generated molecule $M_{g}$and target molecule$M_{t}$, $C\left(M\right)$and$H\left(M\right)$ represent the numbers of carbons and hydrogen in molecule M, and α is a parameter indicating the strength of the penalty. Note that the range of the calculated scores is between 0.0 and 1.0. To calculate the Wasserstein distance between two spectra, the SciPy library was implemented [41]. In the current paper, the term Wasserstein score (WS) is used to refer $Score\left(M_{g},M_{t}\right)$.

### 2.5. Trie enhancement of ChemTS

As mentioned above, ChemTS is a tool that combines a RNN with MCTS. In the context of ChemTS, the MCTS is essentially executed on a prefix search tree. One advantage of ^1^ H NMR spectrum identification is that an enormous number of molecular spectra have been recorded and stored in databases. An intuitive way of utilizing such information is to preload the MCTS prefix search tree with the SMILES strings of the molecules in the database and update the scoring of each traversed node with the WS between the database spectrum and the target spectrum. In computer science, such a prefix tree is usually called trie [42]. See Fig. S2 for an example of trie. We implemented this idea by constructing a trie tree as follows. At every iteration, we inserted one database SMILES string into the trie, on which the nodes were defined in the same way as in ChemTS. After each insertion, the WS of the added SMILES string was used to update the weight of each visited node. The number of preloaded molecules is called the trie size. In our experiments, we tested trie sizes of 0, 1, 100, 1000, and 9800. When the trie size was 1, 100, 1000, and 9800, we ranked the WS for each molecule in the database and selected the top 1, 100, 1000 and 9800 candidates, respectively. The algorithm for trie enhancement is shown in Figure S1.

### 2.6. Database

To show the validity of our concept, we prepared a SMILES database consisting of molecules with relatively small molecular weights. PubChemQC [43], a free and open to the public online database, contains over 3.5 million molecules. Because PubChemQC also provides molecular properties computed by ab initio calculation, the molecular weights of the molecules included in PubChemQC are limited to 500, which was suitable for our purpose.

We downloaded molecules in the form of SMILES strings with PCCDB-IDs from 1 to 138,895. We ran a selection on these 138,895 molecules to pick out the pure organic molecules that consisted of only C, H, N, and O. After selection, 10,548 molecules remained. Eight molecules were removed owing to the failure of the ^1^ H NMR spectrum computation. Charged molecules were also excluded. To verify that molecules not included in the database could be identified using NMR-TS, we removed the test molecules, which are described in the next section, from the database. Finally, 9866 molecules were used as the SMILES database. The database contained the following SMILES characters: O, c, 1, (,), C, =, N, #, n, 2, o, 3, and 4. Since we used a middle-sized database to train our model, the diversity of molecules may not be sufficient to generate certain moieties. For further applications, users may need to retrain the RNN to enhance the performance.

### 2.7. Test set

**Figure 3.**
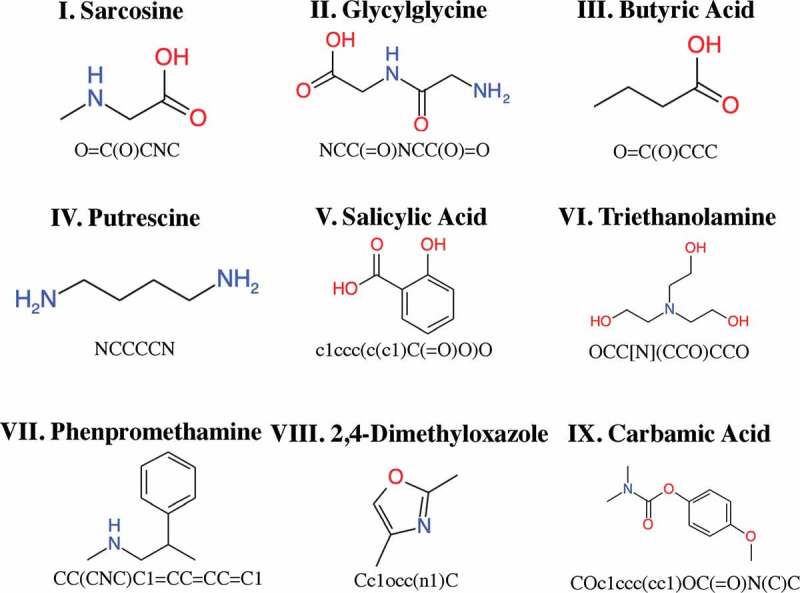
Nine test molecules with their chemical structural formulas and SMILES representations.

We manually selected nine small organic molecules with molecular weights of less than 500 as the test set (Figure 3). We executed parallelized NMR-TS, using 20 cores per execution. The computation time was limited to 100 h for each test molecule. We tested NMR-TS with five different parameter combinations (see Table 1) on the nine test molecules.

**Table 1.** Correct answer rate and average Wasserstein score (WS) for each trie size.

	Target molecules found	Ave. of best WSs
NMR-TS (Trie size = 0)	(1/9)	0.564
NMR-TS (Trie size = 1)	(4/9)	0.778
NMR-TS (Trie size = 100)	(4/9)	0.850
NMR-TS (Trie size = 1000)	(4/9)	0.837
NMR-TS (Trie size = 9800)	(5/9)	0.892
Database search (baseline)	(0/9)	0.740

## 3. Results and discussion

**Figure 4.**
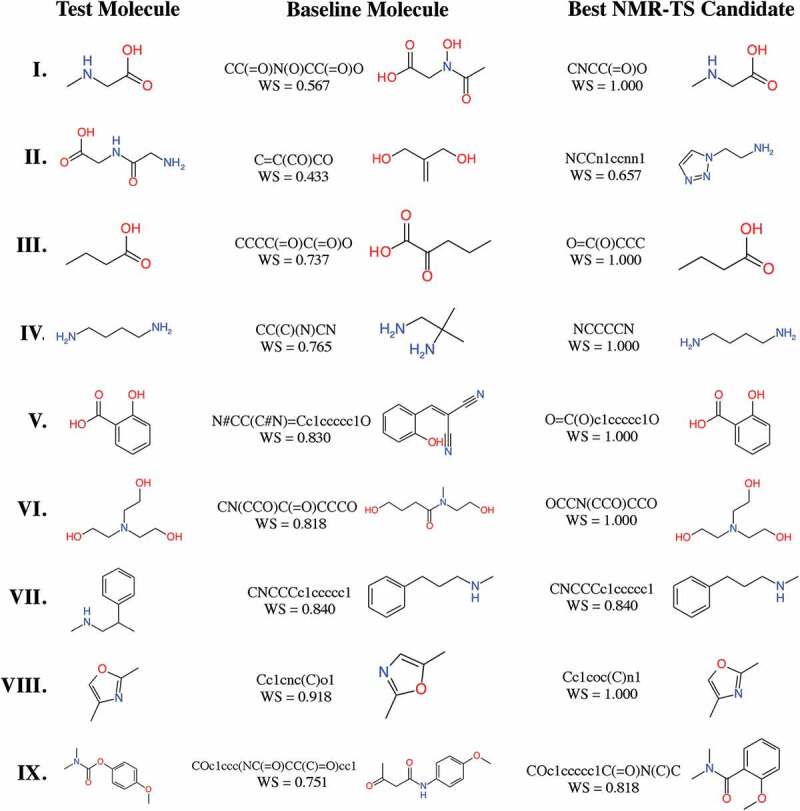
Test molecules, baseline molecules, and best candidate molecules generated by NMR-TS. The corresponding Wasserstein score (WS) is shown for each baseline and candidate molecule. For test molecules **I, III**–**VI**, and **VIII**, NMR-TS gave the correct structures. For test molecules **II, VII**, and **IX**, NMR-TS failed to find the correct structures.

We performed molecule estimation trials by NMR-TS for the test molecules using 20 CPU cores (Intel® Xeon® Gold 6148) for 100 h. For each run, 5451 molecules were generated on average. We defined the molecule in the database with the highest WS as the baseline molecule and its WS as the baseline score. In Table 1, we summarize the correct answer rate and the average WS for each trie size. The baseline molecules and the high-score molecules generated by NMR-TS with various parameters are shown in Figure 4. Since the test molecules were not contained in the database, the baseline molecules had WS values of less than 1.0. As shown in Figure 4, NMR-TS succeeded in identifying six molecules (**I, III, IV, V, VI**, and **VIII**) out of the nine test molecules from their ^1^ H NMR spectra. NMR-TS also suggested other candidates that were close to the target molecules based on the provided spectra.

**Figure 5.**
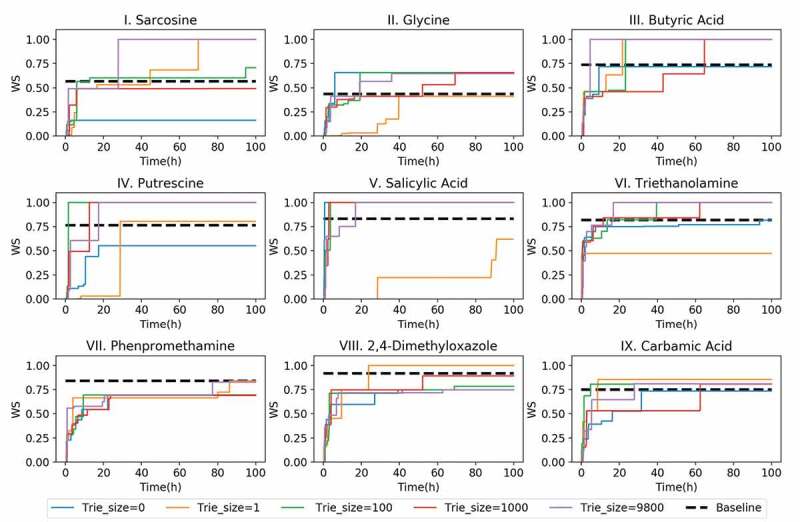
NMR-TS search results for target spectra of test molecules **I**–**IX** showing the best Wasserstein score (WS) as the function of time with different trie sizes. See Table 1 for the details of the different parameter sets.

To demonstrate the dependence of the answer speed on the trie size and the computational time, the evolution of the best WS for each test over time is shown in Figure 5. For all the trie sizes, the WS drastically increased during the initial 10 h. However, when the trie size was zero, NMR-TS could not generate any molecules with a WS of 1.0 within 100 h. Even for the failures (test molecules **II, VII**, and **IX**), NMR-TS succeeded in generating some molecules that had higher WS values than the baseline score when the trie size was increased. See Table S1 for total CPU hours to identify the molecules.

**Figure 6.**
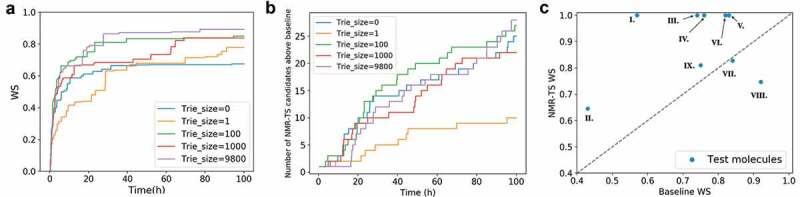
(a) Evolution of the average Wasserstein score (WS) of the best candidates for the nine test molecules over time with different trie sizes. When the trie size is 0, ChemTS starts with a root node without any expansion. When the trie size is 1, 100, 1000, or 9800, a WS is obtained for each spectrum in the database against the target spectrum and based on this ranking, the top 1, 100, 1000, and 9800 molecules, respectively, are fed into the trie. (b) Evolution of the total number of candidates with scores better than the database baseline for all test molecules over time. (c) Comparison of the best candidate scores from the database search and NMR-TS. C = 1, trie size = 9800.

In Figure 6(a), we show the time evolution of the average score of the best candidate for the nine test molecules with different trie sizes. For all the trie sizes, the growth of the WS is mostly saturated within 40 h. Furthermore, NMR-TS typically generated higher-scored candidate molecules when the trie size was increased, with some exceptions.

Figure 6(b) shows the total number of candidates that have higher scores than the baseline molecules as a function of the computational time. For each trie size, the total number of candidates monotonically increased over time. As the trie size increased, NMR-TS generated more candidates with better scores than the baseline. A comparison of the results for trie sizes of 0 and 1 in Figure 6(a,b) reveals that a trie size of 0 was superior to a trie size of 1 from the viewpoint of generating more candidates with better scores than the baseline. In contrast, a trie size of 1 was superior to a trie size of 0 from the viewpoint of generating higher-scored candidates. A reasonable explanation for this phenomenon is that while the trie highlights the most promising branch in the search tree, the presence of the trie also restricts the exploration of other branches and thus reduces the overall diversity.

In Figure 6(c), we show a comparison between the scores of the best NMR-TS candidates and the baseline scores. The points above the diagonal dotted line correspond to cases where NMR-TS found better candidates than the baseline candidate. A score on the vertical axis of 1.0 (**I, III, IV, V**, and **VI**) indicates that NMR-TS succeeded in identifying the exact molecular structure. Although NMR-TS did not reach the baseline score for **VII** and **VIII**, these cases mainly fall on the extreme right side of the horizontal axis, which indicates that a good candidate already existed in the database. On the contrary, in cases where the baseline candidates poorly matched the target molecules (middle to left side of the horizontal axis), NMR-TS surpassed the baseline score.

In the current study, we used ^1^ H NMR peaks from all types of functional groups to verify the concept of identifying chemical structures from only the NMR spectra despite using advance information about the number of carbon and hydrogen atoms. Considering the specificity of ^1^ H NMR spectroscopy, it currently is difficult for NMR-TS to discriminate between hydrogen signals from – NH and – OH groups because their chemical shifts appear in the same range (1–5 ppm). Indeed, for test molecule **II**, NMR-TS completely misidentified the – OH environment as a – NH environment.

Similarly, the proximity of the chemical shifts for alkane protons might prevent NMR-TS from predicting the position of a phenyl group relative to methyl(propyl)amine, as in the case of **VII**. Furthermore, NMR-TS does not currently consider the hyperfine coupling resulting from hydrogen spin–spin coupling. Hence, we expect that NMR-TS will not be able to identify benzene derivatives that are characterized by the positions of substituents (i.e., ortho, meta, and para isomers). For test molecules with possible isomers, NMR-TS successfully identified **V** from its ^1^ H NMR spectrum but failed for **IX** although the position of substituents of the baseline molecule is the same position of the test molecule. We speculate that this result reflects that NMR-TS cannot recognize the position of the substituents on benzene derivatives. Therefore, to improve the accuracy, it might be effective to consider the effect of hyperfine coupling when computing the ^1^ H NMR spectra. However, the computation of the hyperfine coupling constant is time-consuming in electronic structure theory. As an alternative, we are planning to combine the ^1^ H NMR spectrum with other spectra, such as the ^13^ C NMR or ultraviolet visible (UV-vis) spectrum. As a commonly used NMR technique, ^13^ C NMR spectroscopy provides important structural information about organic molecules. Thus, by coupling ^1^ H NMR and ^13^ C NMR spectra, the accuracy of NMR-TS is expected to improve considerably.

## 4. Conclusion and outlook

In this study, we demonstrated NMR-TS, a technique for molecule identification from NMR spectra that combines a de novo molecular generation method with quantum chemical computations. NMR-TS was shown to identify a better molecular structure from a ^1^ H NMR spectrum than the baseline despite receiving less or an equal amount of information. Despite the database not containing any of the test molecule structures, NMR-TS succeeded in utilizing the database information to reach the correct molecules without assistance from a chemist.

When MCTS was originally used in game play, each MCTS simulation consisted of a random playout from the current stage, for which the time cost was small. In the context of NMR-TS, each simulation (playout) required a computationally heavy DFT calculation. To tackle this problem, we designed a trie structure, and the identification performance improved as the size of the prebuilt trie tree increased. This result suggests that (1) NMR-TS generally performs better when incorporating information from an NMR database through a trie structure, and (2) a trie structure could be applied to enhance ChemTS when time-consuming simulations are required and an information database is available.

As a non-knowledge-based method, NMR-TS explores the metric space for a particular target spectrum. We designed the Wasserstein score as the metric in this study. In addition to ^1^ H NMR spectra, data from many other spectroscopic measurements, such as ^13^ C NMR, IR, and UV-vis spectra, could be used as inputs. With a different distance metric, NMR-TS variants can potentially be applied to estimate chemical structures from other chemical properties. It is also possible to take into account other types of measurement techniques such as mass spectrometry, if a distance metric is properly designed.

NMR-TS is still in development and has a handful of limitations and possibilities of improvement. First, SMILES cannot represent many features of organic molecules such as axial chirality. It may be resolved by using graph-based representations [24]. In our study, NMR-TS is tested only with computationally generated spectra and still needs to be tested with experimental spectra where peaks are unclear. Impurities are possible obstacles for accurate identification. NMR-TS cannot identify multiple compounds in a mixture, but could be extended by incorporating peak separation techniques presented in [14]. To save computational time, we employed only one conformer per molecule. If *k* conformers are considered, the accuracy of NMR-TS should improve at expense of almost *k*-fold increase in computational cost. Also, our DFT-based spectrum computation can be replaced, e.g., by ENSO [44] in pursuit of better accuracy. See Fig. S3 for comparison of our spectrum with that of ENSO. ENSO took 250 minutes to compute a spectrum, while our DFT calculation took 11 minutes. Compared to our DFT calculation, ENSO showed better accuracy in predicting the experimental spectrum, presumably because ENSO uses multiple conformers for spectrum calculation, while our calculation relies on only one conformer. For molecule generation from experimental spectra, we would need a robust method like ENSO. At this point, the application of NMR-TS is limited to relatively small molecules due to high computational cost. To deal with larger molecules, the incorporation of fragment assembly [14] into NMR-TS might be beneficial. Finally, it is difficult for users to understand why NMR-TS succeeds for some molecules and fails for others. In general, interpreting the results of a neural-network-based system is known to be very difficult [45]. Nevertheless, some methods for explainable AI might improve the interpretability of NMR-TS [45].

We believe that the NMR-TS concept has various possibilities. For instance, NMR-TS could be utilized for product identification in an automated robotic synthesis system. As the reactants are often known, this information could be incorporated into the molecular generator to constrain the search space and improve the performance. In this study, we focused on the identification of one target molecule. Future work should focus on identifying individual molecules from a spectrum of multiple molecules, as it is often difficult to decide which peaks attribute to each molecule in a sample.

## Supplementary Material

Supplemental Material
