# Supplementary material for: NMR-TS: de novo molecule identification from NMR spectra
Source: Sci Technol Adv Mater. 2020 Jul 30;21(1):552–61. doi: 10.1080/14686996.2020.1793382 (PMC7476483; doi:10.1080/14686996.2020.1793382)
Supplement: Supplemental Material [file TSTA_A_1793382_SM1549.pdf]

Supplementary Material for

NMR-TS: molecule estimation from NMR spectrum using de novo molecule generator

Jinzhe Zhang,*[a,b] Kei Terayama[a,b,c,d] , Masato Sumita[b,e], Kazuki Yoshizoe[b], Kengo Ito[f,g], Jun Kikuchi[f,g,h], and Koji Tsuda[a,b,i]

a. *Graduate School of Frontier Sciences, University of Tokyo, 5-1-5 Kashiwanoha, Kashiwa, Chiba 277-8561, Japan*

b. *RIKEN Center for Advanced Intelligence Project, 1−4−1 Nihonbashi, Chuo City, Tokyo 103-0027, Japan*

c. *Graduate School of Medicine, Kyoto University, Yoshida-konoe-cho, Sakyo-ku, Kyoto, 606-8303 Japan*

d. *RIKEN Medical Sciences Innovation Hub program (MIH), 1-7-22 Suehiro-cho, Tsurumi-ku, Yokohama City, Kanagawa 230-0045, Japan*

e. *NIMS wpi International Center for Materials Nanoarchitectonics (MANA), 1-1 Namiki, Tsukuba, Ibaraki 305-0044, Japan*

f. *RIKEN Center for Sustainable Resource Science, 1-7-22 Suehiro-cho, Tsurumi-ku, Yokohama, Kanagawa 230-0045, Japan*

g. *Graduate School of Medical Life Science, Yokohama City University, 22-2 Seto, Kanazawa Ward, Yokohama, Kanagawa 236-0027, Japan*

h. *Graduate School of Bioagricultural Sciences, Nagoya University, Furo-cho, Chikusa-ku, Nagoya 464-8601, Japan*

i. *NIMS The Center for "Materials research by Information Integration" (CMI2), 1-1 Namiki, Tsukuba, Ibaraki 305-0044, Japan*

Figure S1 Algorithm for Trie enhancement of ChemTS

```
if trieSize > 0 then
    /* Read NMR Database                                                     */
    NMRList, SMILESList ← readTrieDatabase();
    for i ← 0 to len(NMRList) − 1 do
        WSList[i] ← getWassersteinScore(NMRList[i], targetNMR);
        /* Note:  NMRList and targetNMR contains element information of each
           peak(atom), the atom number penalty can therefore be computed     */
    end
    /* Sort and select top-N data points depends on trieSize                 */
    Data ← rowStack(SMILESList, WSList, NMRList);
    sortedData ← sort(data, key = WSList);
    SMILESList ← sortedData[: trieSize, 0];
    /* Create Trie                                                           */
    for i ← 0 to len(SMILESList) − 1 do
        j ← 0;
        state ← ['&'];
        currentNode ← rootNode;
        nodesList ← SMILESToNodes(smiles[i]);
        /* Map database SMILES into prefix search tree                       */
        k ← 0;
        while k < len(nodesList) do
            state[k] ← nodesList[k];
            if nodesList[k] not in currentNode.childNodes then
                newNode ← createNewChildNode(position = nodesList[k], parentNode =
                 currentNode);
                currentNode ← newNode;
            else
                for child in currentNode.childNodes do
                    if child.position == nodesList[k] then
                        currentNode ← child;
                        break;
                    end
                end
            end
            k ← k + 1;
            /* Update traversed nodes with the Wasserstein Score             */
            currentWS ← getWassersteinScore(NMRList[k], targetNMR);
            while currentNode! = None do
                currentNode.Update(currentWS);
                currentNode ← currentNode.parentNode;
            end
        end
    end
end
```

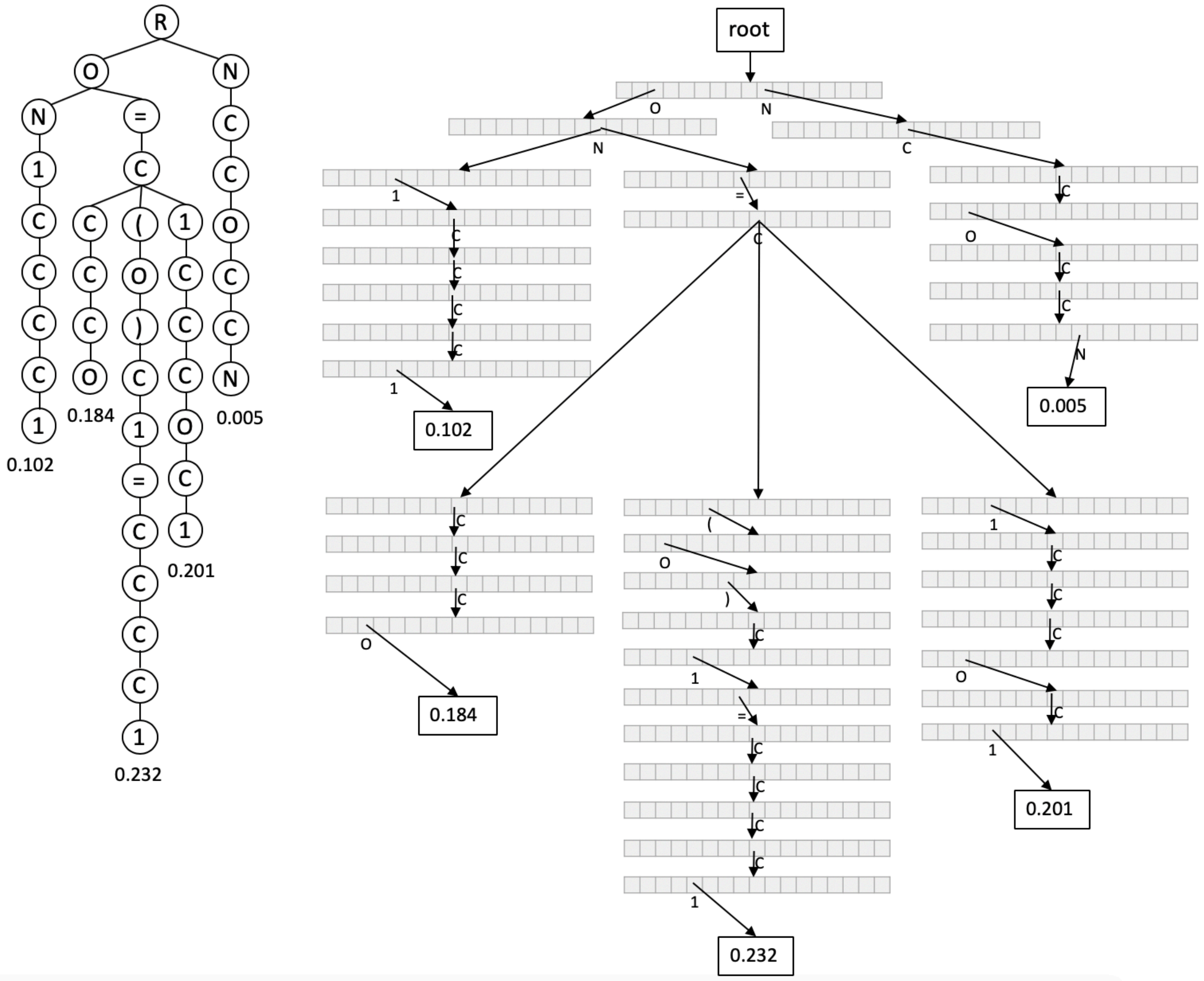

Figure S2 Trie in NMR-TS. Trie is a data structure that represents a set of strings sharing prefixes. A path from the root to a leaf represents one string. The digits at leaf represents the computed score. It is most simply implemented using an array of pointers at each node. The array is looked up with a character, and a next node can be reached by following the found pointer.

Table S1 Estimated CPU Time (hours) for identifying each test molecule.

Trie Size	0	1	100	1000	9800
**I.**	-	1326	-	-	526
**II.**	-	-	-	-	-
**III.**	-	410	441	1229	86
**IV.**	-	-	28	238	330
**V.**	11	-	69	53	320
**VI.**	-	-	750	1182	319
**VII.**	-	-	-	-	-
**VIII.**	-	450	-	-	-
**IX.**	-	-	-	-	-

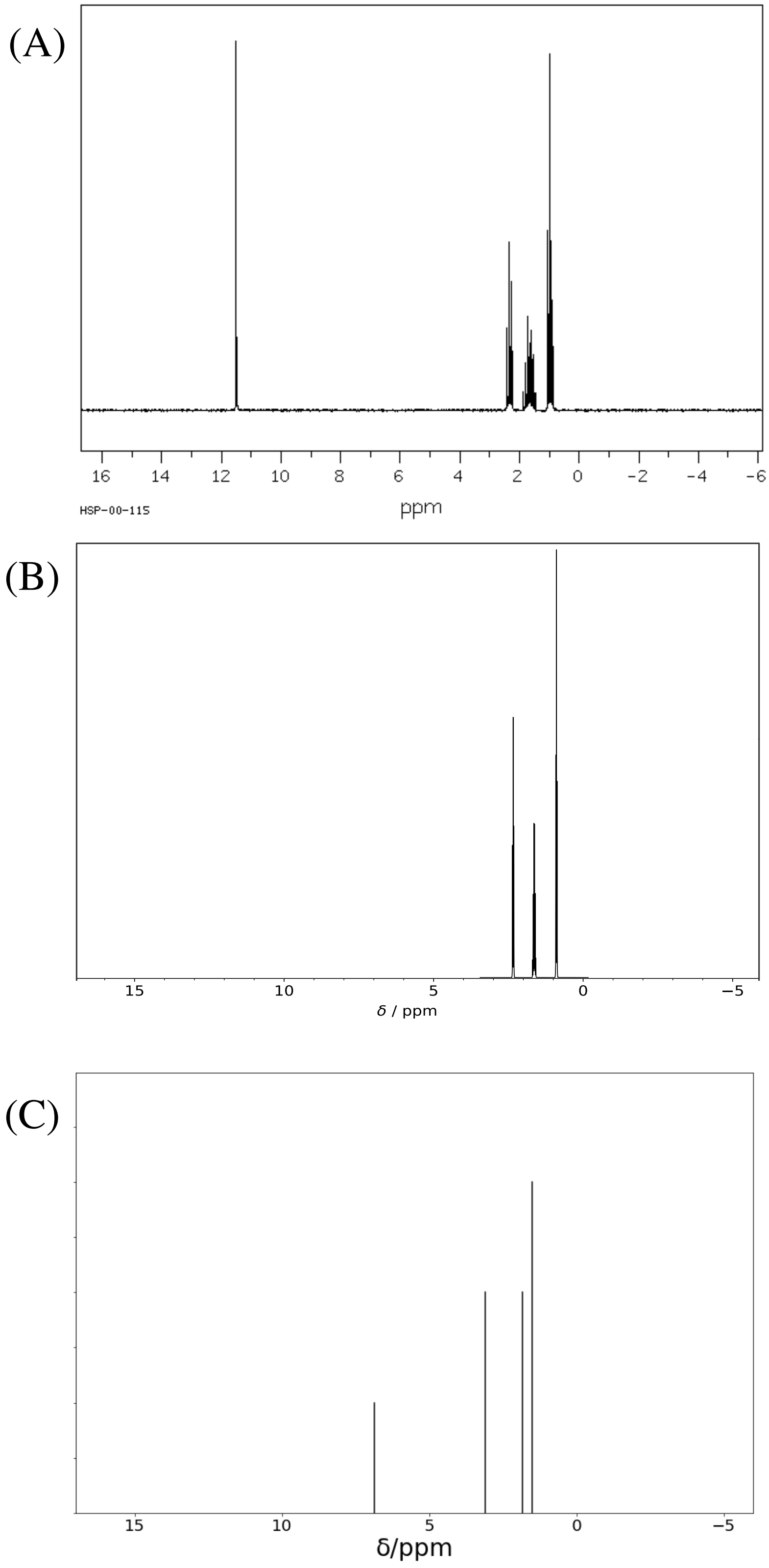

Figure S3 Comparison of **(A)** experiment ^{1}H NMR spectrum obtained from SDBSWeb database, **(B)** ENSO simulated ^{1}H NMR spectrum and **(C)** our DFT method simulated ^{1}H NMR spectrum for Butyric Acid (test molecule **III.**).
